# Caregiving associated with selected cancer risk behaviors and screening utilization among women: cross-sectional results of the 2009 BRFSS

**DOI:** 10.1186/1471-2458-12-685

**Published:** 2012-08-21

**Authors:** Katherine W Reeves, Kathryn Bacon, Lisa Fredman

**Affiliations:** 1 Department of Public Health, University of Massachusetts Amherst, 410 Arnold House, 715 North Pleasant Street, Amherst, MA, 01003, USA; 2 Department of Epidemiology, Boston University, Boston, MA, USA

**Keywords:** Caregiving, Mammography, Pap test, Health behaviors

## Abstract

**Background:**

Informal caregiving is increasingly common as the U.S. population ages, and there is concern that caregivers are less likely than non-caregivers to practice health-promoting behaviors, including cancer screening. We examined caregiving effects on cancer risk behaviors and breast and cervical cancer screening in the 2009 Behavioral Risk Factor Surveillance System.

**Methods:**

Women age ≥41 with data on breast and cervical cancer screening were included (weighted frequency 3,478,000 women). Cancer screening was classified according to American Cancer Society guidelines. We evaluated the association of caregiving with cancer risk behaviors (obesity, physical activity, alcohol intake, smoking status, and fruit/vegetable consumption) and cancer screening (mammography, clinical breast exam [CBE], and Pap test) using logistic regression overall and with stratification on age (<65, ≥65) or race (white, non-white).

**Results:**

Caregivers had greater odds of being obese, physically active, and current smokers. Subgroup analyses revealed that caregiving was associated with obesity in younger women and whites, and with less obesity in older women. Also, caregiving was associated with smoking only among younger women and non-whites. Caregivers had greater odds of ever having had a mammogram or CBE, yet there was no association with mammogram, CBE, or Pap test within guidelines.

**Conclusions:**

Caregiving was associated with some health behaviors that increase cancer risk, yet not with cancer screening within guidelines. Effects of caregiving by age and race require confirmation by additional studies.

## Background

There is recent concern that caregivers are less likely than non-caregivers to practice health-promoting behaviors [1-3], which may include seeking cancer screening tests. Caregivers are generally defined as persons who provide unpaid assistance or supervision with personal or instrumental activities of daily living (i.e., bathing, eating, dressing, medication management, handling finances) to a relative or friend who cannot perform these tasks due to cognitive, physical, or psychological impairments [4]. The increasing number of older caregivers in the United States [5], and their chronically high levels of self-reported stress and potential for adverse health consequences [6], underscores the importance of health maintenance and disease prevention in this population. Receipt of appropriate cancer screening tests is an important component of health maintenance, yet little is known about the cancer screening practices of caregivers as compared to non-caregivers.

Of the estimated 43.5 million U.S. caregivers in 2009, 54% were aged 50 years or older, and 26% spent ≥20 hours/week performing caregiving activities [5]. As the majority (67%) of caregivers are female [5], the health effects of caregiving are an increasingly important women’s health issue. Caregivers consistently report more psychological distress, such as depressive symptoms and anxiety, as well as self-reported stress than non-caregivers [6]. According to the Caregiver Stress Process model, stress may result from characteristics of the caregiving situation, including the care recipient’s condition, level of care required, the necessity to balance caregiving responsibilities with other employment and family responsibilities, and the availability of social support and other factors that may reduce caregiving-related stress, leading to adverse effects on psychological or physical health [7]. As a result of this stress, or a way to cope with caregiving demands, caregivers may engage in risky health behaviors, such as cigarette smoking and alcohol consumption. Likewise, they may neglect health promotion activities, such as regular cancer screening, due to feeling stressed or the time constraints of caregiving [1,2,8]. However, there is conflicting evidence that caregivers have an increased risk of physical health decline or mortality [4,9,10]. In fact, older women caregivers had fewer medical conditions and functional limitations than their non-caregiver peers [4], suggesting that older caregivers may attempt to maintain good health in order to continue providing care to their care recipient.

Scant research has examined the association between caregiving and modifiable health behaviors related to cancer risk or utilization of cancer screening. The results of existing studies are inconsistent [2,3,8,11], with caregivers having some better health habits [2,8,11] and pro-active cancer screening practices [1] as well as some poorer health habits [8]. Likewise, of studies comparing breast and cervical cancer screening behaviors, one reported increased utilization among caregivers [3], while the other reported no association [1]. Most studies to date have been limited by focusing on spousal caregivers [1,3,8], restriction to persons older than age 50 [1,11] or 65 [2,8], not stratifying by gender [1-3,8,11], and lack of adjustment for covariables [2]. Thus, the results of these studies may not relate to cancer-screening guidelines that are generally gender- and age-specific.

Effective screening methods are widely available for breast and cervical cancers, two of the most common cancers among middle-aged and older women. Among U.S. women, an estimated 209,060 incident cases of breast cancer and 12,200 incident cases of cervical cancer were expected in 2010 [12], with the highest incidence rates among women age ≥40 [7]. Though cervical cancer guidelines have recently been revised [13], the American Cancer Society (ACS) previously recommended Pap tests either yearly or every two to three years depending on personal history of Pap test results for women age ≥21 to screen for cervical cancer and annual clinical breast exams (CBE) and mammograms for women age ≥40 to screen for breast cancer [12].

Given the increases in the aging population and resultant need for informal caregiving, it is important to understand whether caregiving is associated with cancer risk and screening behaviors, especially among women. Therefore, we used data from female respondents to the 2009 Behavioral Risk Factor Surveillance System (BRFSS) to examine the association between caregiving and modifiable health behaviors known to affect overall cancer risk, such as smoking, alcohol use, obesity, physical activity, and fruit/vegetable consumption, and on breast and cervical cancer screening. We hypothesized that caregivers would engage in more negative health behaviors and be less likely to receive recommended cancer screenings as compared to non-caregivers. We additionally sought to examine if associations between caregiving and health behaviors and cancer screening differed by age or race. If caregiving is associated with poorer health behaviors or lack of screening utilization, this might identify an opportunity for intervention, as caregivers are likely to have frequent contacts with the health care system due to their caregiving responsibilities.

## Methods

### Study population

Publicly available BRFSS data were obtained from the National Center for Health Statistics website [14]. The BRFSS is an annual telephone survey of health behaviors among U.S. adults age ≥18. Households are selected through random digit dialing and sampling weights are employed to allow for inference to be made to the state and U.S. populations. Each state utilizes the same core questionnaire and may add additional modules. In 2009, Georgia, Hawaii, Tennessee, and Wyoming included a Women’s Health Module that asked female participants questions about breast and cervical cancer screening. We restricted our analysis to women age ≥41 (weighted N = 4,183,000; unweighted N = 12,174) from these four states in order to select the population of women for whom the ACS recommendations included both mammography and a Pap test within the past year. We further excluded participants missing data for caregiving status (weighted N = 70,000; unweighted N = 149) or reporting a history of cancer other than non-melanoma skin cancer (weighted N = 701,000; unweighted N = 2149). The resulting final weighted sample size was 3,478,000 (unweighted N = 10,015).

### Caregiving status

Caregiving status was assessed by one question: “People may provide regular care or assistance to a friend or family member who has a health problem, long-term illness, or disability. During the past month, did you provide any such care or assistance to a friend or family member?”

### Cancer risk behaviors

Body mass index (BMI) was calculated from participants’ self-reported current height and weight. Individuals with values below the 1^st^ percentile or above the 99^th^ percentile of BMI were excluded. The remaining individuals were categorized as normal weight (<25.0 kg/m^2^), overweight (25 - <30 kg/m^2^), or obese (≥30 kg/m^2^). Participants reported their total minutes per week of non-work-related moderate and vigorous physical activity. These measures were combined to generate a total weekly physical activity variable, categorized as 0 minutes/week, 1 – 149 minutes/week, or ≥150 minutes/week. Alcohol use was ascertained by participants’ report of having consumed any alcohol within the past 30 days, the number of days alcohol was consumed during this period, and the average number of drinks consumed each time. Women were classified as non-drinkers (no alcohol consumption), low or moderate drinkers (less than one drink per day), or heavy drinkers (at least one drink per day) using a calculated variable included in the BRFSS dataset. Participants were categorized as never, former, or current smokers based on their report of ever having smoked ≥100 cigarettes in their lifetime and if they currently smoked cigarettes every day, some days, or not at all. A dichotomous measure of daily fruit and vegetable consumption (<5 or ≥5 servings per day) was derived from reported intake of fruit juice, fruit, green salad, potatoes, carrots, and other vegetables; this calculated variable was included in the BRFSS dataset.

### Cancer screening behaviors

Participants reported if they had ever had a mammogram, and if “yes” the length of time since their most recent mammogram. Women whose mammogram was within the past year were classified as receiving a mammogram within ACS guidelines in place at the time of the 2009 BRFSS [12]. Participants were also asked if they had ever had a CBE; those who answered “yes” and whose CBE was within the past year were classified as receiving a CBE within ACS guidelines. Women were classified as receiving breast cancer screening within guidelines if they had both a mammogram and a CBE within the past year.

Participants were asked if they had ever received a Pap test, and the length of time since their most recent Pap test. Because data were not available on history of Pap test results, we were unable to define receiving such screening within guidelines exactly following the ACS definition. Women whose Pap test was within the past three years were classified as receiving cervical cancer screening within guidelines. We also classified women according to whether they had received a Pap smear within the past year or ever.

### Covariables

Sociodemographic variables included self-reported age in years, race (White non-Hispanic, Black non-Hispanic, Other non-Hispanic [i.e. Asian, Native Hawaiian or other Pacific Islander, American Indian or Alaskan Native, other race, or multiracial], and Hispanic), educational attainment (did not graduate from high school, graduated from high school, attended college or technical school, and graduated college or technical school), employment status (employed or self-employed, out of work, homemaker, student, retired, and unable to work), income (<$25,000, $25,000- < $50,000, $50,000- < 75,000, and ≥ $75,000), and marital status (married, not married). For stratified analyses, we created dichotomous variables for age (<65 versus ≥ 65 years), to reflect Medicare eligibility, and race (White versus non-White), due to small numbers in racial categories other than White. Three health-related variables were included: health insurance status (yes, no), self-rated general health (excellent or very good, good, fair, and poor), and whether the respondent had a routine medical checkup in the past year (yes, no).

### Statistical analysis

The BRFSS survey uses stratified sampling to collect survey information; all results reported take into account the survey weights and complex sampling frames. We performed bivariate analyses of the associations between sociodemographic characteristics and caregiver status, as well as caregiver status and each of the health promotion and cancer screening variables. Multivariable logistic regression was used to evaluate the association between caregiving status and cancer risk or screening behaviors. Multivariable, multinomial logistic regression was used for outcomes with more than two categories. Model building began with an evaluation of missing data for each covariable; the variable on “income” had a high number of missings (unweighted N = 1631) and was not included. For each outcome (i.e. cancer risk or screening behavior) the initial adjusted model included all sociodemographic and cancer risk behavior variables, then proceeded through backwards selection to retain only covariables significant at p = 0.05 as well as age and caregiver status. Complete-case analysis was used in each model. The logistic regression analyses were repeated with stratification on age or race, and interaction between caregiving status and age or race was formally assessed by testing the significance of an appropriate interaction term added to the multivariable model.

Analyses were performed using SAS version 9.1.3 Service Pack 4 (SAS Institute, Inc., Cary, North Carolina). A two-tailed p-value of <0.05 was considered statistically significant.

## Results

The final study population included 10,015 participants, which corresponds to a weighted frequency of 3,478,220 women. Compared to non-caregivers, caregivers were significantly younger, and had increased odds of being highly educated, employed, and married (Table 1). Caregivers and non-caregivers did not differ on other sociodemographic or health-related factors.

**Table 1:** **Demographic characteristics of population sample, by caregiver status**^**a**^

**Characteristic**^**b**^	**Caregivers**	**Non-caregivers**	**P value**
	**N (%)**	**N (%)**	
Total population	1,198,000 (34.4)	2,280,000 (65.6)	
Age, years; Mean (SE)	55.9 (0.34)	58.4 (0.26)	<0.0001
41-64	946,000 (79.0)	1,596,000 (70.0)	<0.0001
≥65	252,000 (21.0)	684,000 (30.0)	
Race/ethnicity			0.45
White, non-Hispanic	826,000 (69.2)	1,619,000 (71.7)	
Black, non-Hispanic	231,000 (19.4)	420,000 (18.6)	
Other, non-Hispanic	105,000 (8.8)	163,000 (7.2)	
Hispanic	32,000 (2.7)	57,000 (2.5)	
Education			0.0004
Did not graduate high school	97,000 (8.1)	288,000 (12.6)	
Graduated high school	375,000 (31.3)	740,000 (32.5)	
Attended college or technical school	364,000 (30.4)	611,000 (26.9)	
Graduated college or technical school	361,000 (30.2)	636,000 (28.0)	
Employment status			0.007
Employed or self-employed	590,000 (49.2)	1,031,000 (45.3)	
Out of work	80,000 (6.7)	114,000 (5.0)	
Homemaker	164,000 (13.7)	287,000 (12.6)	
Student	6,000 (0.5)	13,000 (0.6)	
Retired	271,000 (22.6)	614,000 (27.0)	
Unable to work	87,000 (7.3)	217,000 (9.5)	
Income			0.18
<$25,000	313,000 (31.5)	613,000 (32.7)	
$25,000 - < $50,000	272,000 (27.4)	454,000 (24.2)	
$50,000 - < $75,000	135,000 (13.6)	298,000 (15.9)	
≥$75,000	274,000 (27.6)	508,000 (27.1)	
Marital status			<0.0001
Married	813,000 (68.1)	1,393,000 (61.3)	
Not married	380,000 (31.9)	881,000 (38.7)	
Health insurance			0.16
Yes	1,047,000 (87.5)	2,033,000 (89.3)	
No	150,000 (12.5)	243,000 (10.7)	
Self-rated general health			0.09
Excellent/Very good	593,000 (49.5)	1,101,100 (48.4)	
Good	380,000 (31.7)	665,000 (29.3)	
Fair	156,000 (13.0)	342,00 (15.0)	
Poor	69,000 (5.8)	165,000 (7.3)	
Routine medical checkup in past year			0.55
Yes	962,000 (81.1)	1,843,000 (82.0)	
No	224,000 (18.9)	404,000 (18.0)	

^a^Data reported are the weighted frequencies (rounded to the nearest 1000) and percentages, based on an unweighted sample size of 10,015.

^b^The unweighted numbers of missing observations for each variable are as follows: age, 0; race/ethnicity, 91; education, 15; employment status, 27; income, 1631; marital status, 29; health insurance, 18; self-rated general health, 25; routine medical checkup in past year, 158.

### Cancer risk behaviors

Caregivers had greater odds of obesity than non-caregivers, though they also had greater odds of reporting ≥150 minutes of total physical activity each week (Table 2). Caregivers were at somewhat greater odds of being current smokers and consuming more fruits and vegetables each day, though these relationships did not achieve statistical significance. There was no association between caregiving status and alcohol consumption.

**Table 2:** **Cancer risk behaviors of population sample, by caregiver status**^**a**^

**Behavior**^**b**^	**Caregivers**	**Non-caregivers**	**P value**
	**N (%)**	**N (%)**	
Body mass index, kg/m^2^; Mean (SE)	28.0 (0.19)	27.3 (0.11)	0.01
Normal (<25.0 kg/m^2^)	385,000 (35.0)	811,000 (38.9)	0.08
Overweight (25 - <30 kg/m^2^)	377,000 (34.3)	696,000 (33.4)	
Obese (≥30 kg/m^2^)	337,000 (30.7)	577,000 (27.7)	
Total physical activity			0.0006
0 minutes/week	208,000 (17.9)	522,000 (23.8)	
1 – 149 minutes/week	319,000 (27.5)	565,000 (25.8)	
≥150 minutes/week	635,000 (54.6)	1,103,000 (50.4)	
Alcohol use			0.13
None	811,000 (68.3)	1,610,000 (71.5)	
Low or moderate	339,000 (28.5)	574,000 (25.5)	
Heavy	38,000 (3.2)	68,000 (3.0)	
Smoking status			0.07
Never smoker	693,000 (58.1)	1,398,000 (61.6)	
Former smoker	291,000 (24.4)	538,000 (23.7)	
Current smoker	209,000 (17.5)	334,000 (14.7)	
Fruit/vegetable intake			0.06
<5 servings per day	846,000 (70.6)	1,674,000 (73.7)	
≥5 servings per day	352,000 (29.4)	598,000 (26.3)	

^a^Data reported are the weighted frequencies (rounded to the nearest 1000) and percentages, based on an unweighted sample size of 10,015.

^b^The unweighted numbers of missing observations for each variable are as follows: body mass index, 479; physical activity, 335; alcohol use, 105; smoking status, 41; fruit/vegetable intake, 27.

In multivariable logistic regression analyses on the Total Population sample, adjusted for age, employment status, self-rated general health, and the other cancer risk behaviors, caregivers had about 30% increased odds of being obese, physically active, and current smokers (Table 3). In the model using the Total Population sample, there was a statistically significant interaction between caregiving status and age on the outcome of body mass index (p_interaction_ = 0.05). Among older women, caregivers had lower odds than non-caregivers of being obese, while among younger women caregivers had greater odds of being both overweight and obese. Caregiving was not statistically significantly associated with alcohol or fruit and vegetable consumption in multivariable analyses. There was a borderline significant interaction between caregiving status and age on the outcome of alcohol consumption (p_interaction_ = 0.09), with older caregivers having greater odds than non-caregivers of reporting low/moderate alcohol consumption.

**Table 3:** Estimated multivariable odds ratios for association of caregiving status with cancer screening behaviors, overall and stratified by age or race

**Outcome**	**OR (95% CI) for Caregivers compared to Non-caregivers**^**a**^				
	**Total Population**	**Age <65**	**Age ≥65**	**White**	**Non-White**
Body mass index^b^					
Overweight vs. Normal	1.18 (0.98 – 1.41)	1.25 (1.00 – 1.56)	0.90 (0.66 – 1.21)	1.12 (0.91 – 1.37)	1.32 (0.91 – 1.91)
Obese vs. Normal	1.26 (1.02 – 1.54)	1.43 (1.12 – 1.82)	0.65 (0.43 – 0.98)	1.30 (1.02 – 1.64)	1.15 (0.76 – 1.73)
Total physical activity					
1 – 149 minutes/week vs. 0 minutes/week	1.36 (1.08 – 1.72)	1.45 (1.09 – 1.94)	1.03 (0.71 – 1.50)	1.42 (1.08 – 1.85)	1.24 (0.80 – 1.94)
≥150 minutes/week vs. 0 minutes/week	1.30 (1.05 – 1.61)	1.25 (0.95 – 1.65)	1.40 (1.01 – 1.94)	1.42 (1.10 – 1.82)	1.08 (0.70 – 1.66)
Alcohol use^b,c^					
Low or moderate vs. None	0.99 (0.82 – 1.20)	1.06 (0.85 – 1.31)	0.62 (0.44 – 0.90)	0.91 (0.73 – 1.13)	1.21 (0.83 – 1.74)
Heavy vs. None	0.92 (0.61 – 1.38)	0.95 (0.60 – 1.49)	0.70 (0.29 – 1.70)	0.92 (0.58 – 1.45)	0.88 (0.34 – 2.27)
Smoking status^c^					
Former smoker vs. Never smoker	1.16 (0.96 – 1.39)	1.16 (0.92 – 1.46)	1.11 (0.84 – 1.47)	1.07 (0.86 – 1.32)	1.47 (1.04 – 2.08)
Current smoker vs. Never smoker	1.33 (1.06 – 1.67)	1.32 (1.02 – 1.71)	1.29 (0.87 – 1.92)	1.15 (0.88 – 1.49)	2.04 (1.31 – 3.17)
Fruit/vegetable intake					
≥5 servings per day vs. <5 servings per day	0.89 (0.75 – 1.06)	0.87 (0.71 – 1.07)	0.94 (0.71 – 1.25)	0.90 (0.74 – 1.09)	0.85 (0.60 – 1.21)

^a^Odds ratios estimated using multinomial logistic regression and unconditional logistic regression and adjusted for age, employment status, self-rated general health and the other cancer risk behaviors evaluated using appropriate weighting procedures.

^b^P value for age X caregiving interaction term <0.10.

^c^P value for race X caregiving interaction term <0.10.

Stratification by race revealed that the positive associations between caregiving and obesity and physical activity were restricted to whites only, although formal tests revealed no statistically significant interactions (p_interaction_ ranged from 0.26-0.81). Conversely, an interaction between race and smoking status was statistically significant (p_interaction_ = 0.01), with caregiving positively associated with former or current cigarette smoking only among non-whites but not among whites.

### Cancer screening behaviors

Caregivers and non-caregivers were generally similar in their cancer screening behaviors, and no statistically significant differences were observed for receiving either breast or cervical cancer screening within guidelines (Table 4). However, caregivers had significantly greater odds of ever having had a CBE and having had a Pap test within the past year, and showed a non-statistically significant increased tendency toward ever having had a mammogram.

**Table 4:** **Cancer screening behaviors of population sample, by caregiver status**^**a**^

**Screening Exam**^**b**^	**Caregivers**	**Non-caregivers**	**P value**
	**N (%)**	**N (%)**	
Breast cancer screening within guidelines^c^	698,000 (66.7)	1,215,000 (65.3)	0.49
Mammogram within guidelines^d^	761,000 (69.5)	1,413,000 (70.0)	0.76
Ever had a mammogram	1,099,000 (93.1)	2,029,000 (91.2)	0.07
Clinical breast exam within guidelines^e^	812,000 (73.7)	1,431,000 (71.1)	0.36
Ever had a clinical breast exam	1,105,000 (93.8)	2,007,000 (90.6)	0.002
Cervical cancer screening within guidelines^f^	928,000 (84.0)	1,716,000 (82.3)	0.24
Pap test within past year	699,000 (63.3)	1,235,000 (59.2)	0.03
Ever had a Pap test	1,116,000 (95.0)	2,111,000 (95.0)	0.94

^a^Data reported are the weighted frequencies (rounded to the nearest 1000) and percentages, based on an unweighted sample size of 10,015.

^b^The unweighted numbers of missing observations for each variable are as follows: breast cancer screening within guidelines, 1610; mammogram within guidelines, 939; ever had a mammogram, 160; clinical breast exam within guidelines, 1134; ever had a clinical breast exam, 192; cervical cancer screening within guidelines, 780; Pap test within past year, 780; ever had a Pap test, 181.

^c^Received a mammogram and a clinical breast exam within the past year.

^d^Received a mammogram within the past year.

^e^Received a clinical breast exam within the past year.

^f^Received a Pap test within the past three years.

In multivariable logistic regression analyses (Table 5), caregivers had about 40% greater odds of ever having had a mammogram or a CBE. No statistically significant associations were observed with other cancer screening behaviors. Results were generally similar when stratified by age. The increased likelihood of ever having had a CBE was statistically significant only among older caregivers in an age-stratified main effects model, but a formal test of interaction between age and ever had a CBE was not statistically significant (p_interaction_ = 0.95). We observed a borderline significant interaction between race and ever had a CBE (p_interaction_ = 0.08), with a significant association between caregiving and ever had a CBE apparent only among whites. Caregiving was also significantly associated with ever had a mammogram only among whites, although the test of the interaction between race and ever had a mammogram was not statistically significant (p_interaction_ = 0.68).

**Table 5:** **Estimated multivariable odds ratios for association of caregiving status with cancer screening behaviors, overall and stratified by age or race**^**a**^

**Outcome**^**a**^	**OR (95% CI) for Caregivers compared to Non-caregivers**				
	**Total Population**	**Age <65**	**Age ≥65**	**White**	**Non-White**
Breast cancer screening within guidelines^b^	1.05 (0.88 – 1.26)	1.00 (0.99 – 1.02)	1.18 (0.88 – 1.58)	1.17 (0.96 – 1.43)	0.83 (0.58 – 1.19)
Mammogram within guidelines^b^	0.98 (0.82 – 1.18)	0.93 (0.75 – 1.15)	1.10 (0.82 – 1.49)	1.09 (0.88 – 1.34)	0.79 (0.55 – 1.12)
Ever had a mammogram^b^	1.35 (0.98 – 1.86)	1.36 (0.94 – 1.97)	1.06 (0.55 – 2.02)	1.33 (0.92 – 1.92)	1.39 (0.78 – 2.49)
Clinical breast exam within guidelines^b^	1.07 (0.88 – 1.30)	1.05 (0.83 – 1.33)	1.13 (0.84 – 1.54)	1.06 (0.86 – 1.31)	1.06 (0.71 – 1.58)
Ever had a clinical breast exam^b^	1.42 (1.06 – 1.90)	1.32 (0.90 – 1.92)	1.67 (1.08 – 2.58)	1.52 (1.08 – 2.14)	1.27 (0.75 – 2.15)
Cervical cancer screening within guidelines ^c^	1.09 (0.87 – 1.36)	1.12 (0.85 – 1.47)	1.00 (0.72 – 1.38)	1.15 (0.90 – 1.48)	0.84 (0.53 – 1.34)
Pap test within past year ^c^	1.15 (0.97 – 1.36)	1.21 (0.98 – 1.50)	0.97 (0.73 – 1.28)	1.18 (0.97 – 1.43)	1.04 (0.74 – 1.46)
Ever had a Pap test ^c^	0.80 (0.57 – 1.12)	0.81 (0.52 – 1.28)	0.75 (0.56 – 1.21)	0.75 (0.51 – 1.11)	0.79 (0.42 – 1.48)

^a^Odds ratios estimated using unconditional logistic regression with appropriate weighting procedures.

^b^Each outcome is reported as “yes” versus “no”.

^c^Main effects model adjusted for age in years, race/ethnicity, marital status, education, employment status, health insurance status, self-rated general health, regular medical checkup in past year, smoking status, physical activity, and alcohol use. Models for White and Non-white populations include all predictors except for race/ethnicity.

^d^Main effects model adjusted for age in years , race/ethnicity, education, employment status, health insurance status, self-rated general health, regular medical checkup in past year, smoking status, physical activity, BMI, and alcohol use. Models for White and Non-white populations include all predictors except for race/ethnicity.

## Discussion

In this cross-sectional analysis of the 2009 BRFSS, we found that caregiving was associated with behaviors that increase cancer risk (i.e. obesity and smoking) as well as those that reduce cancer risk (i.e. physical activity). Further, caregiving was positively associated with ever having had a mammogram or CBE, but were generally similar in their usage of other cancer screening tests. The associations with obesity, smoking, and physical activity varied by age and race, although tests of interaction terms were not always statistically significant. The association between caregiving and ever having had a mammogram or CBE was observed among whites but not non-whites, though no statistically significant interaction was found.

Most prior studies have reported no differences in health behaviors such as smoking or alcohol use between caregivers and non-caregivers [3,8,11], though these studies generally included only spousal caregivers while our analysis was not restricted to spouse caregivers but did not specify the caregivers’ relationship to the care recipient. Caregiving was associated with increased physical activity in some [4], but not all previous [3,8,11] studies. Another study of BRFSS respondents [2] reported that caregivers were more likely to meet guidelines for weekly physical activity, but did not differ from non-caregivers on fruit and vegetable consumption, smoking and alcohol use, or BMI. This previous study only included individuals age ≥65 and did not examine men and women separately [2], though evidence suggests that caregiving may have different effects for men and women [5]. The suggestion of age and race differences we noted is novel and requires confirmation by additional studies.

Previous studies of caregiving and cancer screening tended to rely on convenience samples and lacked comparison groups of non-caregivers [15-17]. Of studies that compared caregivers to non-caregivers, one reported increased breast and cervical cancer screening in unadjusted analyses of spousal caregivers of cancer patients in South Korea [3], though these results may have reflected having a spouse with cancer more than the experience of caregiving. By contrast, the other study observed no association between caregiving and these screening tests in a nationally representative U.S. sample [1]. We were unable to examine the reasons for our finding that caregivers were more likely to have had mammography or CBE in their lifetime but did not differ on screening within ACS guidelines. One might hypothesize that caregivers were formerly more vigilant about screening exams, yet their caregiving responsibilities diminished their ability to be screened at the recommended frequency.

It is important to note that we relied on cancer screening guidelines published by the American Cancer Society in 2009 [18] to determine whether or not women had received appropriate cancer screening. Other organizations, such as the U.S. Preventive Services Task Force, also make screening recommendations to the public. We chose to use a single set of guidelines for simplicity and consistency, though screening recommendations may differ across organizations. Further, breast and cervical cancer screening guidelines have changed recently [13,19], so our findings may not be generalizable to current cancer screening guidelines.

Our study had several limitations. First, the BRFSS included a single question on caregiving status, and lacked details on the caregiving situation and duration. Thus the caregivers in our study likely represent a wide range of caregiving intensity, reason for caregiving, and familial relationship to the care recipient. Cancer risk and screening behaviors of caregivers may vary by amount of strain associated with caregiving or by the health and the relationship between the care recipient and caregiver. In fact, one study found that caregivers’ health promotion and screening behaviors declined 2–5 years after a spouse’s diagnosis, and then improved after 5 years post-diagnosis [3]. Our results may have revealed significant differences between caregivers and non-caregivers if we were able to use a stricter definition of caregiving, such as requiring caregivers to be assisting the care recipient with one or more ADL/IADL tasks. Further, women who were caregivers in the past but not currently, and who may have changed their health and screening behaviors as a result of caregiving, were counted as non-caregivers in our analysis. This classification might minimize the differences between groups, thus attenuating true associations. Additionally, we were unable to exclude individuals who were care recipients, and these individuals would be classified as non-caregivers in our analysis. This misclassification would likely serve to attenuate any true differences between caregivers and non-caregivers. Further, other modifiable breast and/or cervical cancer risk factors, such as hormone therapy and condom use, were not assessed, nor was history of Pap test results, which is necessary for ACS guidelines. Our definition of meeting guidelines as having a Pap test within the previous three years is likely to be overly conservative as many women may have histories for which more frequent screening is recommended. Finally, the Women’s Health Module was administered in only four states, which may not be generalizable to the population of U.S. women.

However, our study is strengthened by the high quality of the BRFSS, its sampling procedures, and large racially/ethnically diverse sample. The few previous studies on the association between caregiving and cancer risk and screening behaviors have focused on older caregivers. Our age- and race-stratified results make an important contribution to this literature.

## Conclusions

In summary, our results demonstrate that middle-aged and older women caregivers are more likely than non-caregivers to engage in health behaviors that increase, as well as some that reduce, cancer risk. Although receipt of screening tests for breast and cervical cancer was similar between caregivers and non-caregivers, approximately one-third of caregivers had not been screened for breast cancer following ACS guidelines. Thus interventions to reduce cancer risk and mortality among caregivers should focus on reducing obesity and smoking and increasing frequency of mammography and CBE, depending on both age and race. Given their frequent contacts with the health care system due to their roles as caregivers, this is a population subgroup that may be especially amenable to such interventions [6].

## Abbreviations

ACS: American cancer society; BMI: Body mass index; CBE: Clinical breast exam; BRFSS: Behavioral risk factor surveillance system.

## Competing interests

The authors declare that they have no competing interests.

## Authors' contributions

KR participated in the design of the study and helped to draft the manuscript. KB obtained the data, performed the statistical analyses, and helped to draft the manuscript. LF participated in the design of the study and helped to draft the manuscript. All authors read and approved the final manuscript.

## Pre-publication history

The pre-publication history for this paper can be accessed here:

http://www.biomedcentral.com/1471-2458/12/685/prepub
